# Tuberculosis survivors and the risk of cardiovascular disease: analysis using a nationwide survey in Korea

**DOI:** 10.3389/fcvm.2024.1364337

**Published:** 2024-08-09

**Authors:** Jiyoul Yang, Sun-Hyung Kim, Jae Kyeom Sim, Seonhye Gu, Jeong Won Seok, Dae-Hwan Bae, Jun Yeun Cho, Ki Man Lee, Kang Hyeon Choe, Hyun Lee, Bumhee Yang, Kyung Hoon Min

**Affiliations:** ^1^ Division of Pulmonary and Critical Care Medicine, Department of Internal Medicine, Chungbuk National University Hospital, Chungbuk National University College of Medicine, Cheongju, Republic of Korea; ^2^ Division of Pulmonary, Allergy, and Critical Care Medicine, Department of Internal Medicine, Korea University Guro Hospital, Korea University College of Medicine, Seoul, Republic of Korea; ^3^ Department of Epidemiology and Health Informatics, Korea University, Seoul, Republic of Korea; ^4^ Department of Internal Medicine, Chungbuk National University Hospital, Chungbuk National University College of Medicine, Cheongju, Republic of Korea; ^5^ Department of Cardiology, Chungbuk National University College of Medicine, Chungbuk National University College of Medicine, Cheongju, Republic of Korea; ^6^ Division of Pulmonary Medicine and Allergy, Department of Internal Medicine, Hanyang University College of Medicine, Seoul, Republic of Korea

**Keywords:** tuberculosis, cardiovascular disease, 10-year atherosclerotic cardiovascular disorder risk, TB survivors, nationwide database

## Abstract

**Background:**

Although the association between tuberculosis (TB) and cardiovascular disease (CVD) has been reported in several studies and is explained by mechanisms related to chronic inflammation, few studies have comprehensively evaluated the association between TB and CVD in Korea.

**Methods:**

Using the Korea National Health and Nutrition Survey, we classified individuals according to the presence or absence of previous pulmonary TB was defined as the formal reading of a chest radiograph or a previous diagnosis of pulmonary TB by a physician. Using multivariable logistic regression analyses, we evaluated the association between the 10-year atherosclerotic cardiovascular disorder (ASCVD) risk and TB exposure, as well as the 10-year ASCVD risk according to epidemiological characteristics.

**Results:**

Among the 69,331 participants, 4% (*n* = 3,101) had post-TB survivor group. Comparing the 10-year ASCVD risk between the post-TB survivor and control groups, the post-TB survivor group had an increased 10-year ASCVD risk in the high-risk group (40.46% vs. 24.00%, *P* < 0.001). Compared to the control group, the intermediate- and high-risk groups had also significantly increased 10-year ASCVD risks (odds ratio [OR] 1.14, 95% confidence interval [CI] 1.04–1.23 and OR 1.69, 95% CI 1.59–1.78, respectively) in the post-TB survivor group. In the association of CVD among post-TB survivors according to epidemiologic characteristics, age [adjusted OR (aOR) 1.10, 95% CI 1.07–1.12], current smoking (aOR 2.63, 95% CI 1.34–5.14), a high family income (aOR 2.48, 95% CI 1.33–4.62), diabetes mellitus (aOR 1.97, 95% CI 1.23–3.14), and depression (aOR 2.06, 95% CI 1.03–4.10) were associated with CVD in the post-TB survivor group.

**Conclusions:**

Our study findings suggest a higher 10-year ASCVD risk among TB survivors than healthy participants. This warrants long-term cardiovascular monitoring and management of the post-TB population.

## Background

Tuberculosis (TB) is the leading cause of mortality due to infectious diseases and is among the top 10 leading causes of death worldwide (1). With global efforts, such as the implementation of the End TB Strategy, the survival rate after TB treatment is increasing (2), and the long-term management of TB survivors is expected to become more important.

TB causes chronic inflammation and immune activation, leading to the development of atherosclerosis and other cardiovascular conditions, even after TB treatment. Cardiovascular diseases (CVDs) are caused by TB-induced direct cardiac involvement, such as pericardial TB. However, increases in CVD incidence in patients with TB have been reported (3–7). For example, in Taiwan, a large population-based retrospective study found that patients with a history of TB had an approximately 3-fold increased risk of developing CVD compared to those without TB (8). Likewise, another study conducted in Taiwan found that TB is associated with an increased risk of ischemic stroke (9). In addition to the mechanistic effect of chronic inflammation on CVD, the increased CVD risk in patients with TB may be related to factors such as socioeconomic status, lifestyle factors, and comorbidities including diabetes mellitus (DM) and hypertension, which are associated with both TB and CVD (10, 11).

Despite the importance of this increased cardiovascular risk, no study has focused on the association between TB and atherosclerotic cardiovascular disorder (ASCVD) in Korea. We did not find any study that reported a correlation between TB and the 10-year ASCVD risk other than the actual CVD rate. Therefore, using a nationwide surveillance system, we analyzed the 10-year ASCVD risk of post-TB survivors compared to that of participants not affected by TB.

## Methods

### Study population

**Figure 1:**
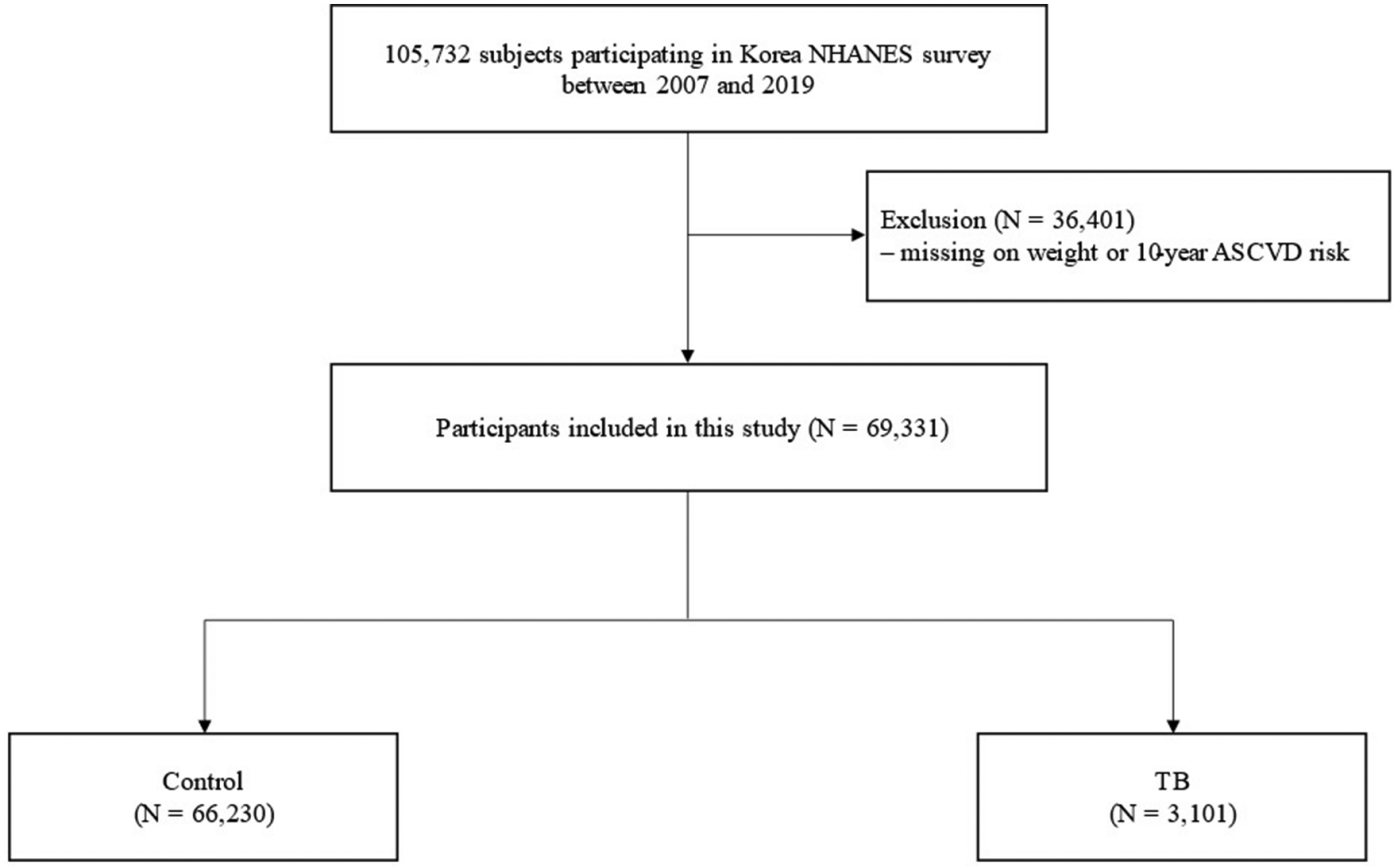
Flow chart. ASCVD, atherosclerotic cardiovascular disorder; NHANES, National health and nutrition examination survey; TB, pulmonary tuberculosis.

The Korea National Health and Nutrition Survey (KNHANES) is a population-based nationwide surveillance system maintained by the Korea Disease Control and Prevention Agency since 1998 to assess the health and nutritional status of Koreans. We used data from KNHANES IV (2007–2009), V (2010–2012), VI (2013–2015), VII (2016–2018), and VIII (2019). The study population was selected using a stratified multistage sampling method. During the 13 years of the study period, 105,732 participants without age limitations were enrolled. Among these, participants with missing weight variables or 10-year ASCVD (*n* = 36,401) were excluded; thus, 69,331 participants were included in this study. The eligible participants were classified into two groups according to their previous TB diagnosis. Previous pulmonary TB was defined as the formal reading of a chest radiograph or a previous diagnosis of pulmonary TB by a physician (Figure 1). The study protocol was approved by the Institutional Review Board of Chungbuk National University Hospital (application no.2023-05-001). The KNHANES were approved by the relevant institutional review boards and all participants provided informed consent.

### Measurements

Available data on age, sex, weight circumference, body mass index (BMI), smoking history, alcohol history, marital status, income status, and educational status were collected from the Korea NHANES database. BMI was calculated as weight in kilograms divided by height in meters squared, and categorized according to Asian-specific criteria: underweight (<18.5 kg/m^2^), normal weight (18.5–22.9 kg/m^2^), overweight (23.0–24.9 kg/m^2^), and obese (≥25.0–29.9 kg/m^2^) (12). Participants who consumed more than 30 g/day of alcohol were classified as heavy drinkers. Comorbidities [asthma, stroke, chronic obstructive pulmonary disease (COPD), DM, hypertension, dyslipidemia, gout, thyroid disease, anemias, chronic kidney disease, depression, family history of stroke and history of cancer] were defined based on self-reported diagnoses by a physician. DM was defined as a fasting glucose level ≥126 mg/dl, the current use of antidiabetic medications, or a self-reported diagnosis of DM by a physician (13). Hypertension was defined as a self-reported diagnosis by a physician, the use of antihypertensive medication, a systolic blood pressure ≥140 mmHg, or a diastolic blood pressure ≥90 mmHg (14). Dyslipidemia was defined as a self-reported diagnosis by a physician, the use of lipid-lowering medication, a total cholesterol concentration ≥240 mg/dl, or a fasting triglyceride concentration ≥200 mg/dl (15).

### Assessment of the 10-year ASCVD risk

CVD is a major cause of disease and death worldwide (16). In 1976, the Framingham Heart Study revealed multiple risk factors and developed for the first time coronary heart disease risk equations (17). Since then, various equations for CVD risk calculation have been published by public health studies and used in clinical practice. Among these, the 10-year ASCVD risk published by the American Heart Association is the most frequently used (18) and allows the calculation of the 10-year risk of cardiovascular problems, such as heart attack or stroke. This risk estimate considers age, sex, race, cholesterol levels, blood pressure, medication use, DM, and smoking status. The ASCVD risk score is expressed as a percentage. A 0%–4.9% risk is considered low, a 5.0%–7.4% risk is considered borderline, a 7.5%–20% risk is considered intermediate, and a risk greater than 20% is considered high (18).

### Outcomes

The main objective of our study was to investigate the correlation between CVD risk and prior TB diagnosis. We also evaluated epidemiological factors associated with CVD in patients with TB.

### Statistical analysis

All analyses were performed using the survey commands in STATA 15.1 version (StataCorp LP, College Station, TX, USA) to account for the complex sampling design and survey weights. All data are presented as weighted percentages with standard errors. Data were compared using Student's *t*-test for continuous variables and Pearson's *χ*^2^ test for categorical variables. Multivariate logistic regression analyses were performed to evaluate the association between TB and CVD. Adjusted odds ratios (aORs) and 95% confidence intervals (CIs) were estimated after adjusting for potential confounding factors (age, BMI, alcohol consumption, marital status, family income, education, and comorbidities). Variables used to calculate the 10-year ASCVD risk (age, sex, race, blood pressure, medication use, DM status, and smoking status) were not adjusted for in the multivariable analysis to minimize collinearity. All tests were two-sided, and *P*-values < 0.05 were considered to indicate significant differences.

## Results

### Baseline characteristics

The baseline characteristics of the participants are presented in Table 1. Of the 69,331 participants, approximately 4% (*n* = 3,101) had TB (post-TB survivor group), and 96% (*n* = 66,230) had never had TB (control group). Compared with the control group, the post-TB survivor group had an older mean age (53.73 years vs. 45.35 years), a higher proportion of male participants (60.20% vs. 49.53%), a higher proportion of underweight participants (6.58% vs. 4.33%), a higher proportion of smokers (53.84% vs. 44.60%), a lower proportion of unmarried participants (10.01% vs. 23.01%), a lower proportion of high familial income (25.88% vs. 30.55%), and a lower level of education (*P* < 0.001 for all variables). Regarding comorbidities, the post-TB survivor group had a higher proportion of participants with asthma (5.75% vs. 2.76%), stroke (2.33% vs. 1.46%), COPD (2.41% vs. 0.33%), DM (14.04% vs. 10.32%), hypertension (35.16% vs. 26.48%), CVD (5.11% vs. 3.11%), liver cirrhosis (0.64% vs. 0.22%) and history of cancer (4.46% vs. 2.96%, *P* < 0.001 for all variables). The proportion of participants with depression was also higher in the post-TB survivor group (4.66% vs. 3.65%, *P *= 0.012). Moreover, the proportion of participants with dyslipidemia was higher in the post-TB survivor group without reaching statistical significance (53.69% vs. 51.89%, *P* = 0.105).

**Table 1:** General characteristics of the study groups (KNHANES cycle 2007–2019).

	Overall (*n* = 69,331)	Control (*n* = 66,230)	Post-TB survivors (*n* = 3,101)	*P*-value
Age, years	45.68 (0.12)	45.35 (0.12)	53.73 (0.34)	<0.001
Male sex	49.95 (0.20)	49.53 (0.20)	60.20 (1.06)	<0.001
Waist circumference, cm	81.69 (0.06)	81.71 (0.06)	81.22 (0.21)	0.023
BMI, kg/m^2^				<0.001
Underweight	4.42 (0.10)	4.33 (0.10)	6.58 (0.53)	
Normal	62.42 (0.23)	62.14 (0.24)	69.30 (0.99)	
Overweight/obesity	33.16 (0.24)	33.54 (0.24)	24.12 (0.93)	
Smoking history				<0.001
Never smoked	55.03 (0.23)	55.40 (0.24)	46.16 (1.10)	
Past smoker	20.66 (0.18)	20.29 (0.19)	29.76 (1.00)	
Current smoker	24.30 (0.24)	24.31 (0.24)	24.08 (1.00)	
Alcohol consumption				0.324
Non-drinker	9.70 (0.15)	9.67 (0.15)	10.46 (0.60)	
Light-to-moderate drinker	74.43 (0.23)	74.48 (0.23)	73.09 (1.01)	
Heavy drinker	15.87 (0.19)	15.85 (0.20)	16.45 (0.88)	
Exercise	84.53 (0.21)	84.56 (0.21)	83.78 (0.82)	0.334
Marital status				<0.001
Unmarried	22.50 (0.30)	23.01 (0.31)	10.01 (0.77)	
Married	67.24 (0.32)	66.88 (0.33)	75.97 (1.02)	
Widowed/separated/divorced	10.27 (0.17)	10.11 (0.17)	14.02 (0.75)	
Family income				<0.001
Low	15.14 (0.27)	14.94 (0.27)	20.11 (0.86)	
Intermediate	54.49 (0.41)	54.51 (0.41)	54.01 (1.16)	
High	30.36 (0.44)	30.55 (0.44)	25.88 (1.07)	
Education				<0.001
Elementary school	16.26 (0.25)	15.96 (0.25)	23.36 (0.9)	
Middle/high school	47.4 (0.33)	47.45 (0.34)	46.2 (1.11)	
College or higher	36.33 (0.38)	36.58 (0.38)	30.44 (1.05)	
High-sensitivity C-reactive protein, mg/L	1.17 (0.02)	1.16 (0.02)	1.46 (0.11)	0.006
Fasting glucose, mg/dl	98.32 (0.12)	98.26 (0.12)	99.79 (0.46)	0.001
Systolic blood pressure, mmHg	117.25 (0.11)	117.13 (0.11)	119.99 (0.38)	<0.001
Diastolic blood pressure, mmHg	75.99 (0.07)	75.98 (0.07)	76.25 (0.24)	0.263
Heart rate, bpm	57.88 (0.23)	57.69 (0.23)	61.89 (1.21)	0.001
HbA1c, %	5.73 (0.01)	5.73 (0.01)	5.85 (0.02)	<0.001
AST, IU/L	22.73 (0.07)	22.69 (0.07)	23.69 (0.33)	0.003
ALT, IU/L	22.68 (0.1)	22.72 (0.1)	21.92 (0.46)	0.088
GGT, IU/L	34.73 (0.59)	34.61 (0.6)	37.38 (2.49)	0.269
Hemoglobin, g/dl	14.21 (0.01)	14.21 (0.01)	14.18 (0.03)	0.513
Platelet, Thous/ul	257.49 (0.32)	257.81 (0.33)	249.42 (1.46)	<0.001
Comorbidities				
Asthma	2.88 (0.08)	2.76 (0.08)	5.75 (0.51)	<0.001
Stroke	1.49 (0.05)	1.46 (0.05)	2.33 (0.31)	0.001
COPD	0.41 (0.03)	0.33 (0.02)	2.41 (0.36)	<0.001
Diabetes mellitus	10.47 (0.15)	10.32 (0.16)	14.04 (0.76)	<0.001
Hypertension	26.82 (0.26)	26.48 (0.26)	35.16 (1.04)	<0.001
Dyslipidemia	51.96 (0.26)	51.89 (0.26)	53.69 (1.09)	0.105
Cardiovascular disease	3.19 (0.08)	3.11 (0.08)	5.11 (0.46)	<0.001
Gout	0.17 (0.02)	0.17 (0.02)	0.39 (0.14)	0.015
Thyroid disease	3.13 (0.08)	3.13 (0.08)	3.06 (0.33)	0.842
Anemias	7.64 (0.12)	7.6 (0.12)	8.64 (0.59)	0.066
Chronic kidney disease	0.27 (0.02)	0.27 (0.02)	0.35 (0.12)	0.430
Liver cirrhosis	0.23 (0.02)	0.22 (0.02)	0.64 (0.16)	<0.001
Depression	3.69 (0.09)	3.65 (0.09)	4.66 (0.44)	0.012
Family history of stroke	8.62 (0.14)	8.55 (0.14)	10.25 (0.69)	0.009
History of cancer	3.02 (0.07)	2.96 (0.07)	4.46 (0.4)	<0.001

Values are reported as weighted percentages or mean (standard deviation).

BMI, body mass index; KNHANES, Korea National health and nutrition examination survey; TB, pulmonary tuberculosis.

### Comparison of 10-year ASCVD risk between post-TB and control groups

A comparison of the 10-year ASCVD risk between the post-TB survivor and control groups is shown in Table 2. Compared to the control group, the post-TB survivor group had a higher 10-year ASCVD risk than the control group in the high-risk stratum (40.46% vs. 24.00%, *P* < 0.001).

**Table 2:** Comparison of 10-year ASCVD risk between post-TB survivor and control group.

	Overall (*n* = 69,331)	Control (*n* = 66,230)	Post-TB survivors (*n* = 3,101)	*P*-value
10-year ASCVD risk (%)				<0.001
Low (<5)	45.88 (0.32)	46.60 (0.32)	28.28 (1.02)	
Borderline (5–7.4)	8.37 (0.13)	8.41 (0.14)	7.39 (0.60)	
Intermediate (7.5–20)	21.10 (0.19)	20.99 (0.20)	23.87 (0.97)	
High (≥20)	24.65 (0.28)	24.00 (0.28)	40.46 (1.09)	

Values are reported as weighted percentages or mean (standard deviation).

ASCVD, atherosclerotic cardiovascular disorder; TB, pulmonary tuberculosis.

### Association of 10-year ASCVD risk with prior TB diagnosis

**Figure 2:**
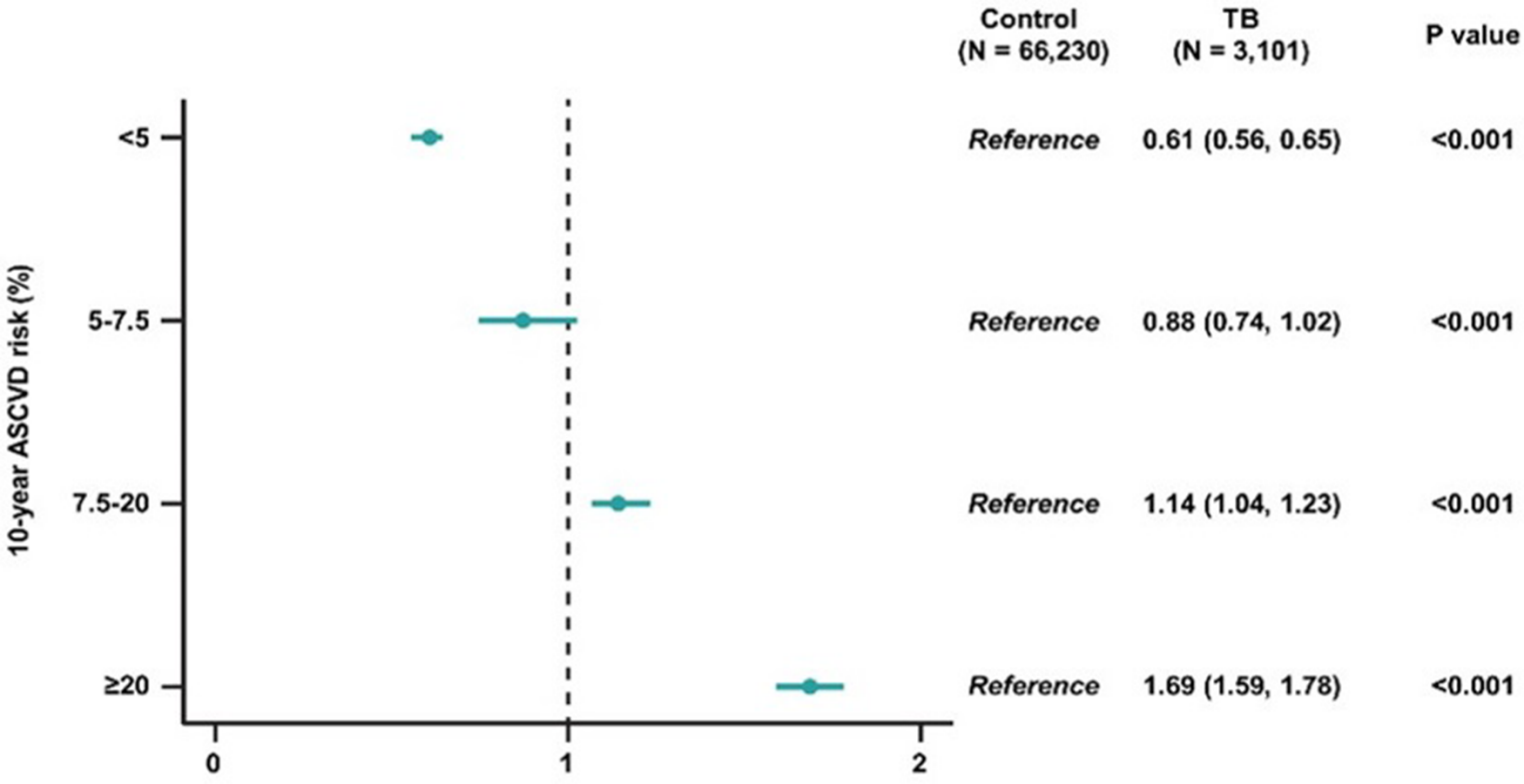
Odds ratios and 95% confidence intervals of 10-year ASCVD risk in post-TB survivors, compared to participants without TB. ASCVD, atherosclerotic cardiovascular disorder; TB, pulmonary tuberculosis.

Figure 2 shows the association between the 10-year ASCVD risk and TB exposure. Compared to the control group, the intermediate and high-risk strata of the post-TB survivor group had significantly higher 10-year ASCVD risks (OR 1.14, 95% CI 1.04–1.23 in the intermediate risk group; OR 1.69, 95% CI 1.59–1.78 in the high-risk group).

### Factors associated with CVD among patients with TB

The multivariable analysis results of CVD association among patients with TB are shown in Table 3. In multivariable analyses, age [adjusted OR (aOR) 1.10, 95% CI 1.07–1.12], current smoker (aOR 2.63, 95% CI 1.34–5.14), high family income (aOR 2.48, 95% CI 1.33–4.62), DM (aOR 1.97, 95% CI 1.23–3.14) and depression (aOR 2.06, 95% CI 1.03–4.10) were factors significantly associated with CVD among patients with TB.

**Table 3:** Multivariable analysis for associations of cardiovascular disease among TB patients.

	Univariable		Multivariable	
	OR (95% CI)	*P* value	OR (95% CI)	*P* value
Age, years	1.08 (1.06, 1.09)	<0.001	1.10 (1.07, 1.12)	<0.001
Male sex	2.07 (1.40, 3.07)	<0.001	1.23 (0.67, 2.26)	0.507
BMI, kg/m^2^		0.003		0.051
Underweight	0.33 (0.12, 0.88)		0.34 (0.12, 0.99)	
Normal	Reference		Reference	
Overweight/obesity	1.60 (1.07, 2.40)		1.38 (0.87, 2.20)	
Smoking history		0.002		0.008
Never-smoker	Reference		Reference	
Past smoker	2.08 (1.39, 3.11)		1.37 (0.75, 2.52)	
Current smoker	1.98 (1.20, 3.27)		2.63 (1.34, 5.14)	
Alcohol consumption		0.194		
Non-drinker	Reference		–	
Light to moderate drinker	0.83 (0.50, 1.35)		–	
Heavy drinker	0.50 (0.23, 1.11)		–	
Marital status		<0.001		0.110
Unmarried	Reference		Reference	
Married	31.99 (5.66, 180.77)		5.40 (0.90, 32.56)	
Widowed/separated/divorced	40.82 (6.95, 239.8)		4.03 (0.60, 26.87)	
Family income		0.009		0.016
Low	Reference		Reference	
Intermediate	0.51 (0.33, 0.77)		1.39 (0.86, 2.22)	
High	0.63 (0.38, 1.05)		2.48 (1.33, 4.62)	
Education		0.001		0.035
Elementary school	Reference		Reference	
Middle/high school	0.77 (0.52, 1.14)		1.48 (0.98, 2.22)	
College or higher	0.35 (0.21, 0.60)		0.77 (0.41, 1.44)	
Comorbidities				
Asthma	2.26 (1.27, 4.02)	0.006	1.75 (0.89, 3.46)	0.106
Diabetes mellitus	4.03 (2.68, 6.07)	<0.001	1.97 (1.23, 3.14)	0.005
Hypertension	3.25 (2.23, 4.73)	<0.001	1.14 (0.74, 1.76)	0.550
Dyslipidemia	2.05 (1.43, 2.93)	<0.001	1.41 (0.94, 2.11)	0.098
Depression	2.15 (1.23, 3.78)	0.008	2.06 (1.03, 4.10)	0.040
COPD	1.48 (0.64, 3.44)	0.362	–	
Chronic kidney disease	6.28 (1.24, 31.72)	0.026	5.24 (0.48, 57.71)	0.176

Values are reported as weighted percentages or mean (standard deviation).

BMI, body mass index; TB, pulmonary tuberculosis; COPD, chronic obstructive pulmonary disease.

## Discussion

We investigated the association between TB survival and CVD risk (defined as the 10-year ASCVD risk) using nationally representative data from Korea. Our study showed that TB survivors had a higher risk of ASCVD than participants without prior TB diagnosis indicating long-term cardiovascular consequences of a TB infection. Additionally, among patients with TB, age, current smoking, high family income, DM and depression were factors significantly associated with CVD.

Previous studies have consistently shown a high risk of CVD development in TB survivors (4, 9, 19, 20). As an example, a meta-analysis conducted in Taiwan showed that patients with TB had a 1.76-fold increased risk of developing coronary heart disease compared to controls (19). In another study, the incidence of ischemic stroke was 1.52 times higher in tuberculosis patients (21). Our study also showed that the post-TB survivor group had a significantly higher 10-year ASCVD risks (OR 1.69, 95% CI 1.59–1.78 in the high-risk group) compared to the control group, aligning with similar results from previous studies. The mechanisms underlying this association are not fully understood; however, one possible mechanism has been proposed (16). TB is known to cause chronic inflammation and immune activation, leading to the development of atherosclerosis and other cardiovascular conditions, even after TB treatment. Possible mechanisms include increased expression of proinflammatory cytokines, monocyte/macrophage immune activation, CD4+ Th1 and Th17 cell immune activation, and autoimmunity mediated by antibodies against mycobacterial hit shock protein 65 (16). Via one or several of these mechanisms, patients surviving TB have a higher incidence of chronic diseases, such as ischemic stroke, CVD, chronic kidney disease, and pulmonary complications, than healthy individuals (7, 22–24).

Our study showed that epidemiologic factors, age, and current smoking were associated with CVD in post-TB survivors. Age (25, 26) and smoking (27–29) are well known as common risk factors for tuberculosis and CVD. Along the same line, it can be interpreted that they are also factors related to the occurrence of CVD in post-TB survivors. Additionally, we showed that a high family income was associated with CVD in post TB-survivors. This finding is contrary to the previous notion that a lower income level is associated with myocardial infarction, sudden cardiac death and the risk of TB (30, 31). Unfortunately, the reason for this phenomenon cannot be fully explained given the observational nature of our study, so further research on this is considered necessary.

Regarding comorbidities, our findings showed that DM and depression were factors significantly associated with CVD in post TB survivors. A systematic review of 13 observational studies reports that DM increases the risk of developing tuberculosis threefold (32). Although the mechanism is not fully understood, it is believed that DM may contribute to TB development due to dysfunctional immune responses (33). Additionally, because DM is also known to be a risk factor for CVD (34), DM is considered a factor associated with CVD in TB survivors. Similarly, the prevalence of CVD in patients with depression is over two-to-three times that in the general population (35). Some mechanisms are biologically plausible for depression to increase the incidence of CVD. These include changes in the autonomic nervous system (36), coagulation factors, such as fibrinogen, and proinflammatory cytokines (37). Patients with depression also tend to have poor adherence to medical treatment. These factors may lead to association between CVD and in patients with depression (38).

The association between TB and the risk of ASCVD has important implications for the long-term management of TB survivors. Considering the increased risk of ASCVD in TB survivors, our findings highlight the need to closely monitor cardiovascular risk factors and provide appropriate interventions, such as control of hypertension and hyperglycemia or smoking cessation to mitigate the risk of TB survivors, especially in those with comorbidities. Further exploration of the underlying mechanisms associated with TB infection and CVD, such as the role of specific inflammatory markers or immune responses, will enhance our understanding of its pathophysiology and guide targeted interventions.

Our study has three major limitations. First, this was a cross-sectional study; thus, the actual CVD risk might not be accurate for TB survivors. Second, this study was performed in a single country; therefore, our results may not be generalizable. Similar studies should be conducted in other countries and ethnicities. Third, in KHANES, it is difficult to identify the various types of ASCVD or CVD that the participants have, such as MI, CAD without MI, PVD, etc (39, 40). However, this study lacks sufficient analysis of the various types of ASCVD or CVD in post-TB survivor. Further research is needed on post-TB survivor.

## Future directions

Future researches should include prospective studies to trace trajectory of the long-term cardiovascular health of TB survivors and investigate specific inflammatory markers linked to increased ASCVD risk in TB survivors for targeted interventions. Finally similar studies should be conducted in diverse populations to generalize findings and understand regional variations in TB related ASCVD risk.

## Conclusion

In conclusion, this study found a higher 10-year ASCVD risk among TB survivors than among control participants, and this finding warrants long-term cardiovascular monitoring and management in this population. Our study suggests that a comprehensive approach that includes early identification and treatment of comorbidities, lifestyle modifications, and cardiovascular risk reduction strategies can help improve the long-term cardiovascular outcomes of TB infection.

## Key messages

1. TB survivors are a higher 10-year ASCVD risk than among control participants.
2. Among TB patients, age, current smoker, high family income, DM and depression were factors significantly associated with CVD.
3. Comprehensive approaches that include the early identification and treatment of comorbidities, lifestyle modifications, and cardiovascular risk reduction strategies can help improve the long-term cardiovascular consequences of TB infection.

## Data Availability

The raw data supporting the conclusions of this article will be made available by the authors, without undue reservation.
