# Supplementary material for: Tuberculosis survivors and the risk of cardiovascular disease: analysis using a nationwide survey in Korea
Source: Front Cardiovasc Med. 2024 Aug 9;11:1364337. doi: 10.3389/fcvm.2024.1364337 (PMC11341360; doi:10.3389/fcvm.2024.1364337)
Supplement: Supplementary file 1 [file Datasheet1.pdf]

```
//*===============================================================================
//* Tuberculosis survivors and the risk of cardiovascular disease: Analysis using a nationwide survey in Korea
//* Running title: ASCVD risk in post TB survivors
//*===============================================================================


//================================================================================
// #1
// creating dataset

use "HN07_19_ALL", clear

d kstrata psu wt_itvex wt_ex1 year ///
HE_tb HE_TB ///
DI4_dg DI4_pt ///
BH9_11 ///
age sex HE_BMI ///
BS1_1 BS3_1 ///
marri_1 marri_2 ///
ho_incm ///
town_t ///
edu ///
BE3_31 ///
HE_tb4 HE_tb5 HE_rPLS ///
EQ5D ///
DE1_dg DE1_31 DE1_32 HE_glu HE_HbA1c ///
HE_sbp HE_dbp DI1_2 DI1_dg ///
DI2_dg DI2_2 HE_TG HE_HDL_st2 ///
DN1_dg DN1_pt HE_crea ///
DI3_dg DI3_pt ///
BD1 BD1_11 BD2_1  ///
HE_chol ///
BO1_2 BP1 GS_mea_r_2 GS_mea_r_3 GS_mea_l_2 GS_mea_l_3 ///
BE3_31 BE3_32 BE3_33 LQ_1EQL ///
DF2_dg DF2_pt ///
DJ4_dg DJ4_pt DJ4_3 ///
DJ2_dg DJ2_pt ///
HE_wc DI3_dg DI2_dg BE3_31 DJ5_dg HE_PFTdr DM8_dg ///
DE2_dg HE_anem HE_hsCRP DN1_dg DK4_dg DF2_dg HE_glu HE_sbp HE_dbp HE_mPLS ///
HE_HbA1c HE_ast HE_alt HE_rGTP HE_HB HE_Bplt ///
HE_STRfh1 HE_STRfh2 HE_STRfh3 DC1_dg ///
DC2_dg DC3_dg DC4_dg DC5_dg DC6_dg DC7_dg DC11_dg

keep kstrata psu wt_itvex wt_ex1 year ///
HE_tb HE_TB ///
DI4_dg DI4_pt ///
BH9_11 ///
```

```
age sex HE_BMI ///
BS1_1 BS3_1 ///
marri_1 marri_2 ///
ho_incm ///
town_t ///
edu ///
BE3_31 ///
HE_tb4 HE_tb5 HE_rPLS ///
EQ5D ///
DE1_dg DE1_31 DE1_32 HE_glu HE_HbA1c ///
HE_sbp HE_dbp DI1_2 DI1_dg ///
DI2_dg DI2_2 HE_TG HE_HDL_st2 ///
DN1_dg DN1_pt HE_crea ///
DI3_dg DI3_pt ///
BD1 BD1_11 BD2_1 ///
HE_chol ///
BO1_2 BP1 GS_mea_r_2 GS_mea_r_3 GS_mea_l_2 GS_mea_l_3 ///
BE3_31 BE3_32 BE3_33 LQ_1EQL ///
DF2_dg DF2_pt ///
DJ4_dg DJ4_pt DJ4_3 ///
DJ2_dg DJ2_pt ///
HE_wc DI3_dg DI2_dg BE3_31 DJ5_dg HE_PFTdr DM8_dg ///
DE2_dg HE_anem HE_hsCRP DN1_dg DK4_dg DF2_dg HE_glu HE_sbp HE_dbp HE_mPLS ///
HE_HbA1c HE_ast HE_alt HE_rGTP HE_HB HE_Bplt ///
HE_STRfh1 HE_STRfh2 HE_STRfh3 DC1_dg ///
DC2_dg DC3_dg DC4_dg DC5_dg DC6_dg DC7_dg DC11_dg

replace HE_tb=HE_TB if year==2013

drop HE_TB

rename *, lower
count
save "data_tb_riskcvd_v01", replace



//========================================================================================
// #2
// generating variables

set more off
use "data_tb_riskcvd_v01", clear

// weight
d wt_itvex wt_ex1 year
codebook wt_itvex wt_ex1 year
sum wt_itvex wt_ex1
tab year, m
*weight-rel
```

```
replace wt_itvex=wt_ex1 if year==2013
tab year, sum(wt_itvex)
tab year, sum(wt_ex1)
*weight-yr
gen wt_ex_pool = wt_itvex*0.5/12.5 if year==2007
replace wt_ex_pool = wt_itvex*1/12.5 if year>2007
codebook wt_ex_pool


// Pulmonary TB
d he_tb dj2_dg
codebook he_tb dj2_dg
tab he_tb dj2_dg, m
tab year he_tb , m

gen tb=0
replace tb=1 if dj2_dg==1 | he_tb ==2
tab tb, m
tab year tb, m row


// Age, years
d age
sum age
codebook age
tab year, sum(age)


// Sex, female
d sex
codebook sex
tab year sex, m row


// BMI, kg/m2
d he_bmi
sum he_bmi
codebook he_bmi
tab year, sum(he_bmi)

gen bmi_cat=0 if he_bmi<18.5
replace bmi_cat=1 if he_bmi>=18.5 & he_bmi<25
replace bmi_cat=2 if he_bmi>=25 & he_bmi!=.
tab bmi_cat, m
tab year bmi_cat, m row

label define bmi_cat 0 "0:Underweight" 1 "1:Normal weight" 2
"2:Overweight/obesity"
label value bmi_cat bmi_cat


// Smoking history
```

```
//   Non-smoker
//   Past smoker
//   Current smoker
tab bs1_1 bs3_1, m
tab year bs1_1, m row
tab year bs3_1, m row

gen smk_status=0 if (bs1_1==3)
replace smk_status=1 if ((bs1_1==1|bs1_1==2) & (bs3_1==3) & (year>=2010)) ///
                       |((bs1_1==1|bs1_1==2) & (bs3_1==2) & (year<2010))
replace smk_status=2 if ((bs1_1==1|bs1_1==2) & (bs3_1==1|bs3_1==2) & (year>=2010)) ///
                       |((bs1_1==1|bs1_1==2) & (bs3_1==1) & (year<2010))
tab smk_status, m
tab year smk_status, m row

label define smk_status 0 "0:Non-smoker" 1 "1:Past smoker" 2 "2:Current smoker"
label value smk_status smk_status

gen smk_status_current=0 if smk_status==0 | smk_status==1
replace smk_status_current=1 if smk_status==2
tab smk_status_current smk_status, m

label define smk_status_current 0 "0:Non-smoker" 1 "1:Current smoker"
label value smk_status_current smk_status_current


// Alcohol consumption
//   Heavy drinker
//   Nonheavy drinker
tab1 bd1 bd1_11 bd2_1, m

gen drk_freq_d=0            if bd1_11==1
replace drk_freq_d=0.5/30.5 if bd1_11==2
replace drk_freq_d=1/30.5   if bd1_11==3
replace drk_freq_d=3/30.5 if bd1_11==4
replace drk_freq_d=2.5/7      if bd1_11==5
replace drk_freq_d=4/7        if bd1_11==6
tab drk_freq_d bd1_11, m

gen drk_amt=1.5 if bd2_1==1
replace drk_amt=3.5 if bd2_1==2
replace drk_amt=5.5 if bd2_1==3
replace drk_amt=8 if bd2_1==4
replace drk_amt=10 if bd2_1==5
tab drk_amt bd2_1, m
```

```
gen drk_amt_freq_w=drk_amt*drk_freq_d*7
sum drk_amt_freq_w
browse drk_amt_freq_w drk_freq_d bd1_11 drk_amt bd2_1

gen drk_status=0 if bd1==1
replace drk_status=1 if drk_freq_d==0 | ///
                        (drk_amt_freq_w<8 & sex==2) | (drk_amt_freq_w<15 & sex==1)
replace drk_status=2 if (drk_amt_freq_w>=8 & drk_amt_freq_w!=. & sex==2) ///
                      | (drk_amt_freq_w>=15 & drk_amt_freq_w!=.& sex==1)

tab drk_status sex, m
tab drk_status drk_freq_d, m
tab sex, sum(drk_amt_freq_w)
tab year drk_status, m row

label define drk_status 0 "0:Non-drinker" 1 "1:Light to moderate drinker " 2 "2:Heavy drinker"
label value drk_status drk_status


// Marital status
//   Unmarried
//   Married
//   Widowed/Separated/Divorced
d marri_1 marri_2
codebook marri_1 marri_2
tab year marri_1, m row
tab year marri_2, m row
tab marri_1 marri_2, m

gen marri_cat=0 if marri_1==2
replace marri_cat=1 if marri_1==1 & marri_2==1
replace marri_cat=2 if marri_1==1 & marri_2==2|marri_2==3|marri_2==4
tab marri_cat, m
tab year marri_cat, m row

label define marri_cat 0 "0:Unmarried" 1 "1:Married" 2 "2:Widowed/Separated/Divorced"
label value marri_cat marri_cat


// Family income
//   Low
//   Intermediate
//   High
d ho_incm
codebook ho_incm
tab year ho_incm, m row
```

```

gen ho_incm_cat=0 if ho_incm==1
replace ho_incm_cat=1 if ho_incm==2 | ho_incm==3
replace ho_incm_cat=2 if ho_incm==4

tab ho_incm_cat ho_incm, m
tab year ho_incm_cat, m row

label define ho_incm_cat 0 "0:Low" 1 "1:Intermediate" 2 "2:High"
label value ho_incm_cat ho_incm_cat


// Education
//   Elementary school graduate
//   Middle/High school graduate
//   College graduate
d edu
codebook edu
tab year edu, m row

gen edu_cat=0 if edu==1
replace edu_cat=1 if edu==2|edu==3
replace edu_cat=2 if edu==4

tab edu_cat, m
tab year edu_cat, m row

label define edu_cat 0 "0:Elementary school" 1 "1:Middle/High school" 2 "2:College"
label value edu_cat edu_cat


// Asthma
d dj4_dg dj4_pt dj4_3

gen ast=0
replace ast=1 if dj4_dg==1 | dj4_pt==1 | dj4_3==1 | dj4_3==2
tab ast, m
tab year ast, m row


// Diabetes mellitus
d de1_dg de1_31 de1_32 he_glu he_hba1c
codebook de1_dg de1_31 de1_32 he_glu he_hba1c
tab de1_dg, m
tab de1_31, m
tab de1_32, m
sum he_glu he_hba1c
tab year, sum(he_glu)
tab year, sum(he_hba1c)

gen dm=0
```

```
replace dm=1 if (he_glu>=126 & he_glu!=.) ///
                |de1_32==1|de1_31==1|de1_dg==1 ///
                |(he_hba1c>=6.5 & he_hba1c!=.)
tab dm, m
tab year dm, m row


// Hypertension
d he_sbp he_dbp di1_2 di1_dg
codebook he_sbp he_dbp di1_2 di1_dg
tab di1_2 di1_dg, m
sum he_sbp he_dbp

gen htn=0
replace htn=1 if (he_sbp>=140 & he_sbp!=.) ///
            |(he_dbp>=90 & he_dbp!=.) ///
            |di1_2==1|di1_2==2|di1_2==3|di1_2==4 ///
            |di1_dg==1
tab htn, m
tab year htn, m row


// Dyslipidemia
d di2_dg  di2_2 he_hdl_st2 he_tg
codebook  di2_dg  di2_2 he_hdl_st2 he_tg
tab di2_dg  di2_2, m
sum he_hdl_st2 he_tg

gen hyp=0
replace hyp=1 if di2_dg==1 ///
            |di2_2==1|di2_2==2|di2_2==3|di2_2==4 ///
            |(he_hdl_st2<40 & sex==1) ///
            |(he_hdl_st2<50 & sex==2) ///
            |(he_tg>=150 & he_tg!=.)
tab hyp, m
tab year hyp, m row


// CVD (Cardiovascular disease)
d di4_dg di4_pt
codebook di4_dg di4_pt
tab year di4_dg, m row
tab year di4_pt, m row

gen cvd=0
replace cvd=1 if di4_dg==1 | di4_pt==1 | di3_dg==1 | di3_pt==1
tab cvd, m
tab year cvd, m row


// Depression
d df2_dg df2_pt
```

```
gen dep=0
replace dep=1 if df2_dg==1 | df2_pt==1
tab dep, m
tab year dep, m row


// 10 year ASCVD risk (%)
codebook sex age he_chol he_hdl_st2 he_sbp smk_status_current dm

gen ascvd_ind=(-29.799)*ln(age) + ///
                4.884*(ln(age))^2 + ///
                13.540*ln(he_chol) + ///
                (-3.114)*ln(age)*ln(he_chol) + ///
                (-13.578)*ln(he_hdl_st2) + ///
                3.149*ln(age)*ln(he_hdl_st2) + ///
                1.957*ln(he_sbp) + ///
                7.574*smk_status_current + ///
                (-1.665)*ln(age)*smk_status_current + ///
                0.661*dm ///
                if sex==2

replace ascvd_ind=12.344*ln(age) + ///
                   11.853*ln(he_chol) + ///
                   (-2.664)*ln(age)*ln(he_chol) + ///
                   (-7.990)*ln(he_hdl_st2) + ///
                   1.769*ln(age)*ln(he_hdl_st2) + ///
                   1.764*ln(he_sbp) + ///
                   7.837*smk_status_current + ///
                   (-1.795)*ln(age)*smk_status_current + ///
                   0.658*dm ///
                   if sex==1
sum ascvd_ind
tab sex, sum(ascvd_ind)
tab year, sum(ascvd_ind)
list sex age he_chol he_hdl_st2 he_sbp smk_status_current dm
ascvd_ind in 1/5


* Revision: C10 - Table 1
d he_wc di3_dg di2_dg be3_31 dj5_dg he_pftdr dm8_dg ///
  de2_dg he_anem he_hscrp dn1_dg dk4_dg df2_dg he_glu he_sbp
he_dbp he_mpls ///
  he_hba1c he_ast he_alt he_rgtp he_hb he_bplt ///
  he_strfh1 he_strfh2 he_strfh3 dc1_dg ///
  dc2_dg dc3_dg dc4_dg dc5_dg dc6_dg dc7_dg dc11_dg


// waist circumference, cm
hist he_wc
tab year, sum(he_wc)
```

```

// stroke
tab di3_dg, m
gen str=0
replace str=1 if di3_dg==1
tab str di3_dg, m
tab year str , m row


// exercise : regular walking
d be3_31
codebook be3_31
tab be3_31, m
tab year be3_31, m row

gen reg_wal=0 if be3_31==1
replace reg_wal=1 if be3_31==2|be3_31==3|be3_31==4|be3_31==5|be3_31==6 ///
                     |be3_31==7|be3_31==8
tab reg_wal, m
tab year reg_wal, m row


// COPD
tab1 dj5_dg he_pftdr, m
gen copd=0
replace copd=1 if dj5_dg==1 | he_pftdr==1
tab year copd, m row


// Gout
tab dm8_dg, m
gen gout=0
replace gout=1 if dm8_dg==1
tab gout dm8_dg, m
tab year gout, m row


// Hypothyroidism : Thyroid disease
tab de2_dg, m
gen hypothy=0
replace hypothy=1 if de2_dg==1
tab hypothy de2_dg, m
tab year hypothy, m row


// Anemias
tab he_anem, m
gen anem=0
replace anem=1 if he_anem==1
tab anem he_anem, m
tab year anem, m row
```

```
// high-sensitivity C-reactive protein, mg/L
sum he_hscrp
tab year, sum(he_hscrp)


// Chronic kidney disease
tab dn1_dg, m
gen ckd=0
replace ckd=1 if dn1_dg==1
tab ckd dn1_dg, m
tab year ckd, m row


// Liver cirrhosis
tab dk4_dg, m
gen livercirr=0
replace livercirr=1 if dk4_dg==1
tab livercirr dk4_dg, m
tab year livercirr, m row


// Fasting glucose, mg/dL
hist he_glu
tab year, sum(he_glu)


// Blood pressure (Systolic), mmHg
hist he_sbp
tab year, sum(he_sbp)


// Blood pressure (Diastolic), mmHg
hist he_dbp
tab year, sum(he_dbp)


// Heart rate, bpm
hist he_mpls
tab year, sum(he_mpls)


// HbA1c (%)
hist he_hba1c
tab year, sum(he_hba1c)


// ALT (IU/L)
hist he_ast
tab year, sum(he_ast)
```

```

// AST (IU/L)
hist he_alt
tab year, sum(he_alt)


// GGT (IU/L)
hist he_rgtp
tab year, sum(he_rgtp)


// Hemoglobin, g/dL
hist he_hb
tab year, sum(he_hb)


// Platelet, Thous/uL
hist he_bplt
tab year, sum(he_bplt)


// Family history of stroke
tab1 he_strfh1 he_strfh2 he_strfh3, m
gen fh_str=0
replace fh_str=1 if he_strfh1==1 | he_strfh2==1 | he_strfh3==1
tab fh_str, m
tab year fh_str, m row


// History of cancer
tab1 dc1_dg dc2_dg dc3_dg dc4_dg dc5_dg dc6_dg dc7_dg dc11_dg, m
gen cancer=0
replace cancer=1 if dc1_dg==1 | dc2_dg==1 | dc3_dg==1 | dc4_dg==1
 | dc5_dg==1 | dc6_dg==1 | dc7_dg==1 | dc11_dg==1
tab cancer, m
tab year cancer, m row


count
save "data_tb_riskcvd_v02", replace



//==================================================================
========================
// #3
// definition of study population

set more off
use "data_tb_riskcvd_v02", clear

tab year, m
```

```
count
// screening population: (N=105,732) subjects participating in Korea NHANES survey between 2007 and 2019

count if missing(wt_ex_pool)
*(N=10,008) missing on weight variable
count if missing(ascvd_ind)
*(N=33,904) missing on weight variable
drop if missing(wt_ex_pool) | missing(ascvd_ind)
*(36,401 observations deleted)
count
// eligible population: (N=69,331) Participants included in this study
tab tb, m
//          tb |      Freq.     Percent        Cum.
// ------------+-----------------------------------
//           0 |     66,230       95.53       95.53
//           1 |      3,101        4.47      100.00
// ------------+-----------------------------------
//       Total |     69,331      100.00




// 10 year ASCVD risk (%) : individual sum -> risk는 study pop
tab sex, sum(ascvd_ind)

//             |        Summary of ascvd_ind
//        성별 |        Mean   Std. Dev.       Freq.
// ------------+------------------------------------
//           1 |    60.31324   1.9681747      30,056
//           2 |  -29.945638   1.7080744      39,275
// ------------+------------------------------------
//       Total |   9.1829024   44.766241      69,331


gen ascvd_mean=-29.945638 if sex==2
replace ascvd_mean=60.31324 if sex==1
tab sex, sum(ascvd_mean)

gen ascvd_risk=1-0.9144^exp(ascvd_ind-ascvd_mean)

gen ascvd_risk_perc=100*ascvd_risk
sum ascvd_risk_perc
tab sex, sum(ascvd_risk_perc)
list sex ascvd_ind ascvd_mean ascvd_risk ascvd_risk_perc in 1/5

*quartiles
sum ascvd_risk_perc, detail

//   Q1 < 2.42
//   Q2 2.42 >=, < 9.64
```

```
//   Q3 9.64 >=, < 30.55
//   Q4 30.55 >=

gen ascvd_risk_perc_cat=0     if ascvd_risk_perc<2.42
replace ascvd_risk_perc_cat=1 if ascvd_risk_perc>=2.42 & ascvd_risk_perc<9.64
replace ascvd_risk_perc_cat=2 if ascvd_risk_perc>=9.64 & ascvd_risk_perc<30.55
replace ascvd_risk_perc_cat=3 if ascvd_risk_perc>=30.55 & ascvd_risk_perc!=.
tab ascvd_risk_perc_cat, m
tab year ascvd_risk_perc_cat, m col
bysort ascvd_risk_perc_cat: sum ascvd_risk_perc

label define ascvd_risk_perc_cat 0 "0:<2.42" 1 "1:2.42-9.64" 2 "2:9.64-30.55" 3 "3:>=30.55"
label value ascvd_risk_perc_cat ascvd_risk_perc_cat



* cut-off 5%
gen ascvd_risk_perc_cat_5=0     if ascvd_risk_perc<5
replace ascvd_risk_perc_cat_5=1 if ascvd_risk_perc>=5 & ascvd_risk_perc!=.
tab ascvd_risk_perc_cat_5, m
tab year ascvd_risk_perc_cat_5, m col
bysort ascvd_risk_perc_cat_5: sum ascvd_risk_perc

label define ascvd_risk_perc_cat_5 0 "0:<5" 1 "1:>=5"
label value ascvd_risk_perc_cat_5 ascvd_risk_perc_cat_5
tab ascvd_risk_perc_cat_5, m


* cut-off 7.5%
gen ascvd_risk_perc_cat_7dot5=0     if ascvd_risk_perc<7.5
replace ascvd_risk_perc_cat_7dot5=1 if ascvd_risk_perc>=7.5 & ascvd_risk_perc!=.
tab ascvd_risk_perc_cat_7dot5, m
tab year ascvd_risk_perc_cat_7dot5, m col
bysort ascvd_risk_perc_cat_7dot5: sum ascvd_risk_perc

label define ascvd_risk_perc_cat_7dot5 0 "0:<7.5" 1 "1:>=7.5"
label value ascvd_risk_perc_cat_7dot5 ascvd_risk_perc_cat_7dot5
tab ascvd_risk_perc_cat_7dot5, m


* cut-off 20%
gen ascvd_risk_perc_cat_20=0     if ascvd_risk_perc<20
replace ascvd_risk_perc_cat_20=1 if ascvd_risk_perc>=20 & ascvd_risk_perc!=.
tab ascvd_risk_perc_cat_20, m
tab year ascvd_risk_perc_cat_20, m col
```

```
bysort ascvd_risk_perc_cat_20: sum ascvd_risk_perc

label define ascvd_risk_perc_cat_20 0 "0:<20" 1 "1:>=20"
label value ascvd_risk_perc_cat_20 ascvd_risk_perc_cat_20
tab ascvd_risk_perc_cat_20, m


*10-year ASCVD risk
*Q1-Q4 cut off <5%, 5-7.5%, 7.5-20%, >=20%
sum ascvd_risk_perc, detail

gen ascvd_risk_perc_cat_57520=0     if ascvd_risk_perc<5
replace ascvd_risk_perc_cat_57520=1 if ascvd_risk_perc>=5 & ascvd_risk_perc<7.5
replace ascvd_risk_perc_cat_57520=2 if ascvd_risk_perc>=7.5 & ascvd_risk_perc<20
replace ascvd_risk_perc_cat_57520=3 if ascvd_risk_perc>=20 & ascvd_risk_perc!=.
tab ascvd_risk_perc_cat_57520, m
tab year ascvd_risk_perc_cat_57520, m col
bysort ascvd_risk_perc_cat_57520: sum ascvd_risk_perc

label define ascvd_risk_perc_cat_57520 0 "0:<5" 1 "1:5-7.5" 2 "2:7.5-20" 3 "3:>=20"
label value ascvd_risk_perc_cat_57520 ascvd_risk_perc_cat_57520


save "data_tb_riskcvd_v03", replace



//========================================================================================
// #4
// descriptive analysis

*Table 1. General characteristics of the study groups (KNHANES cycle 2007-2019)
set more off
use "data_tb_riskcvd_v03", clear

svyset [pweight=wt_ex_pool], strata(kstrata) psu(psu)
svydescribe

tab tb, m

svy: mean age
svy, over(tb): mean age
svy: regress age i.tb


tab1 sex bmi_cat smk_status drk_status marri_cat ho_incm_cat
```

```
edu_cat ast dm htn hyp cvd dep ascvd_risk_perc_cat , m
foreach x of varlist sex bmi_cat smk_status drk_status marri_cat
ho_incm_cat edu_cat ast dm htn hyp cvd dep ascvd_risk_perc_cat {
svy: proportion `x'
}
///
foreach x of varlist sex bmi_cat smk_status drk_status marri_cat
ho_incm_cat edu_cat ast dm htn hyp cvd dep ascvd_risk_perc_cat {
svy, subpop(if tb==0): proportion `x'
svy, subpop(if tb==1): proportion `x'
svy: tab `x' tb
}
///

*5, 7.5, 20
svy: proportion ascvd_risk_perc_cat_57520
svy, subpop(if tb==0): proportion ascvd_risk_perc_cat_57520
svy, subpop(if tb==1): proportion ascvd_risk_perc_cat_57520
svy: tab ascvd_risk_perc_cat_57520 tb
///


*Revision: C10 - Table 1
sum he_wc he_hscrp he_glu he_sbp he_dbp he_mpls he_hba1c he_ast
he_alt he_rgtp he_hb he_bplt
tab1 str reg_wal copd gout hypothy anem ckd livercirr fh_str
cancer, m

foreach x of varlist he_wc he_hscrp he_glu he_sbp he_dbp he_mpls
he_hba1c he_ast he_alt he_rgtp he_hb he_bplt {
    svy: mean `x'
    svy, over(tb): mean `x'
    svy: regress `x' i.tb
}
///
foreach x of varlist str reg_wal copd gout hypothy anem ckd
livercirr fh_str cancer {
    svy: proportion `x'
    svy, subpop(if tb==0): proportion `x'
    svy, subpop(if tb==1): proportion `x'
    svy: tab `x' tb
}

svyset [pweight=wt_ex_pool], strata(kstrata) psu(psu) singleunit(
centered)
svydescribe
svy: mean he_mpls
svy, over(tb): mean he_mpls
svy: regress he_mpls i.tb
```

```
//================================================================
======================
// #5
//

*Table 2. Comparison of 10-year ASCVD risk between post-TB
survivor and control groups

set more off
use "data_tb_riskcvd_v03", clear
svyset [pweight=wt_ex_pool], strata(kstrata) psu(psu)
svydescribe

tab ascvd_risk_perc_cat, m
svy: logit tb i.ascvd_risk_perc_cat, or

svy: mlogit ascvd_risk_perc_cat i.tb , base(0)
margins i.tb, atmeans post
nlcom _b[1. _predict#1. tb] / _b[1. _predict#0. tb]
nlcom _b[2. _predict#1. tb] / _b[2. _predict#0. tb]
nlcom _b[3. _predict#1. tb] / _b[3. _predict#0. tb]
nlcom _b[4. _predict#1. tb] / _b[4. _predict#0. tb]


*5, 7.5, 20
tab ascvd_risk_perc_cat_57520, m
svy: logit tb i.ascvd_risk_perc_cat_57520, or

svy: mlogit ascvd_risk_perc_cat_57520 i.tb , base(0)
margins i.tb, atmeans post
nlcom _b[1. _predict#1. tb] / _b[1. _predict#0. tb]
nlcom _b[2. _predict#1. tb] / _b[2. _predict#0. tb]
nlcom _b[3. _predict#1. tb] / _b[3. _predict#0. tb]
nlcom _b[4. _predict#1. tb] / _b[4. _predict#0. tb]



//================================================================
======================
// #6
//

* Figure 2. Odds ratios and 95% confidence intervals of 10-year
ASCVD risk in post-TB survivors, compared to participants
without TB.
gen age_cat65 = 0
replace age_cat65 = 1 if age >= 65
tab age_cat65, m

replace sex = 0 if sex == 2 /*sex = 1 if male, sex = 2 -> 0 if
female*/

```

```
*Unweighted N
foreach x of varlist age_cat65 sex bmi_cat smk_status drk_status marri_cat ho_incm_cat edu_cat ast dm htn hyp cvd dep {
tab `x' tb
}
///
*Weighted %
foreach x of varlist age_cat65 {
svy: proportion `x'
svy, subpop(if tb==0): proportion `x'
svy, subpop(if tb==1): proportion `x'
svy: tab `x' tb
}
///
*adjusted OR (95% CI)
svy: logit tb i.age_cat65 i.sex ib1.bmi_cat i.smk_status i.drk_status i.marri_cat i.ho_incm_cat i.edu_cat i.ast i.dm i.htn i.hyp i.cvd i.dep, or


coefplot, drop(_cons) xline(1) eform yscale(alt axis(2)) ///
    group( ///
    1.age_cat65 =                   "1,160  25.76   1.48 (1.28, 1.71)" ///
    1.sex =                         "1,774  60.2    1.86 (1.63, 2.13)" ///
    0.bmi_cat =                     "210    6.58    1.88 (1.57, 2.27)" ///
    2.bmi_cat =                     "738    24.12   0.56 (0.50, 0.62)" ///
    1.smk_status =                  "956    29.76   1.15 (1.00, 1.32)" ///
    2.smk_status =                  "632    24.08   0.90 (0.77, 1.05)" ///
    1.drk_status =                  "2,253  73.09   1.01 (0.87, 1.17)" ///
    2.drk_status =                  "438    16.45   1.05 (0.86, 1.28)" ///
    1.marri_cat =                   "2,396  75.97   2.59 (2.16, 3.1)" ///
    2.marri_cat =                   "488    14.02   3.08 (2.45, 3.87)" ///
    1.ho_incm_cat =                 "1,561  54.01   0.99 (0.87, 1.13)" ///
    2.ho_incm_cat =                 "723    25.88   0.91 (0.78, 1.06)" ///
    1.edu_cat =                     "1,328  46.2    0.93 (0.81, 1.07)" ///
    2.edu_cat=                      "843    30.44   0.83 (0.70, 0.97)" ///
    1.ast =                         "198    5.75    2.05 (1.68, 2.50)" ///
```

```
    1.dm =                              "467    14.04   1.05 (0.92, 1.21)" ///
    1.htn =                             "1,206  35.16   1.14 (1.02, 1.27)" ///
    1.hyp =                             "1,712  53.69   1.02 (0.92, 1.12)" ///
    1.cvd =                             "196    5.11    0.97 (0.79, 1.19)" ///
    1.dep =                             "169    4.66    1.27 (1.03, 1.56)", angle(horizontal)) ///
    coeflabel( ///
    1.age_cat65 = "Age≥65 vs. <65" ///
    1.sex = "Male vs. Female" ///
    0.bmi_cat = "BMI: Underweight vs. Normal" ///
    2.bmi_cat = "BMI: Overweight/obesity vs. Normal" ///
    1.smk_status = "Smoking history: Past smoker vs. Never-smoker" ///
    2.smk_status = "Smoking history: Current smoker vs. Never-smoker" ///
    1.drk_status = "Alcohol consumption: Light to moderate drinker vs. Non-drinker" ///
    2.drk_status = "Alcohol consumption: Heavy drinker vs. Non-drinker" ///
    1.marri_cat = "Marital status: Married vs. Unmarried" ///
    2.marri_cat = "Marital status: Widowed/Separated/Divorced vs. Unmarried" ///
    1.ho_incm_cat= "Female income: Intermediate vs. Low" ///
    2.ho_incm_cat = "Female income: High vs. Low" ///
    1.edu_cat= "Education: Middle/High school vs. Elementary school" ///
    2.edu_cat = "Education: College or higher vs. Elementary school" ///
    1.ast = "Asthma" 1.dm = "Diabetes mellitus" 1.htn = "Hypertension" 1.hyp = "Dyslipidemia" 1.cvd = "Cardiovascular disease" 1.dep = "Depression")



//===========================================================================================
// #7
//

* Table 3. Multivariable analysis for associations of cardiovascular disease among TB patients
tab tb cvd, m

svyset [pweight=wt_ex_pool], strata(kstrata) psu(psu)

svy, subpop(if tb == 1): logit cvd age, or

foreach x of varlist /*age*/ sex /*bmi_cat*/ smk_status
```

```
drk_status marri_cat ho_incm_cat edu_cat ast dm htn hyp dep {
    svy, subpop(if tb == 1): logit cvd i.`x', or
    svy, subpop(if tb == 1): tab cvd `x'
}
///
svy, subpop(if tb == 1): logit cvd ib1.bmi_cat, or
svy, subpop(if tb == 1): tab cvd bmi_cat

* Revision : COPD, CKD
svy, subpop(if tb == 1): logit cvd i.copd, or
svy, subpop(if tb == 1): logit cvd i.ckd, or


svy, subpop(if tb == 1): logit cvd age i.sex ib1.bmi_cat i.smk_status /*i.drk_status*/ i.marri_cat i.ho_incm_cat i.edu_cat i.ast i.dm i.htn i.hyp i.dep /*i.copd*/ i.ckd, or
testparm i.bmi_cat
testparm i.smk_status
testparm i.marri_cat
testparm i.ho_incm_cat
testparm i.edu_cat
```
